# Current Epidemiological Status of Chikungunya Virus Infection in East Africa: A Systematic Review and Meta-Analysis

**DOI:** 10.1155/2024/7357911

**Published:** 2024-10-25

**Authors:** Abdirasak Sharif Ali Mude, Yahye Ahmed Nageye, Kizito Eneye Bello

**Affiliations:** ^1^Department of Microbiology and Laboratory Science, Faculty of Medicine and Health Sciences, SIMAD University, Mogadishu 252, Somalia; ^2^Department of Microbiology, Faculty of Natural Science, Kogi State (Prince Abubakar Audu) University, Anyigba PMB 1008, Kogi State, Nigeria

**Keywords:** chikungunya, East Africa, epidemiology, meta-analysis, prevalence, PRISMA, systematic review

## Abstract

**Background:** The incidence of Chikungunya in tropical Africa is still of major epidemiological significance. This study aims to determine the prevalence of chikungunya in East Africa through a systematic review and meta-analysis of published studies.

**Methods:** We conducted a comprehensive search across six electronic databases—Web of Science, PubMed, ScienceDirect, Scopus, and Google Scholar—using specific keywords to address the worldwide impact of chikungunya following the Preferred Reporting Items for Systematic Reviews and Meta-analysis (PRISMA) guidelines. A meta-analysis was performed on our eligible studies using the random effect model.

**Results:** Our search returned 40 eligible articles involving 4122 Chikungunya cases in 13 East African nations. These studies, conducted between 2014 and 2024 across 13 East African nations, provided diverse data on chikungunya prevalence. The overall pooled prevalence of chikungunya in East Africa was 20.6% (95% CI: 18.8%–22.5% and *I*^2^ = 99.62%). Subgroup analyses revealed variations in prevalence across different countries, study designs, detection methods, and publication years. Notably, Rwanda and Djibouti exhibited high prevalence rates of 63.0% and 50.4%, respectively, while Kenya and Somalia reported a moderate prevalence of 12.2%. The detection methods also influenced prevalence rates, with RT-PCR studies indicating a higher prevalence (28.3%) compared to ELISA (19.3%).

**Conclusion:** The study highlights the significant burden of chikungunya in East Africa, and the findings underscore the need for targeted public health interventions and improved surveillance to manage and control chikungunya outbreaks in the region.

## 1. Introduction

Chikungunya, a viral disease transmitted by mosquitoes, has attracted considerable worldwide attention because of its substantial impact on public health [1]. The disease, which presents significant health risks, is caused by the chikungunya virus (CHIKV) from the Togaviridae family [2, 3]. The clinical manifestations of numerous arboviral infections, such as chikungunya, are nearly impossible to differentiate. The main mode of transmission is by the Aedes mosquito species, which can transmit the virus both horizontally and vertically [4, 5]. The initial documentation of CHIKV occurred in 1952, after an epidemic of dengue-like disease was observed among the Makonde population in southern Tanzania [6]. The virus subsequently disseminated to further areas within sub-Saharan Africa [7]. The initial occurrence of the outbreak outside of Africa was documented in Bangkok, Thailand, making Asia the second location to observe its appearance. While chikungunya fever was not considered a significant worry until it reemerged in 2005-2006, there have since been multiple recorded outbreaks of the disease worldwide [3, 8]. The lack of accurate reporting and misdiagnosis have hindered the establishment of dependable yearly global data on chikungunya cases.

The presence of typical symptoms of chikungunya, as well as the long-lasting chronic arthritis and cognitive impairments associated with the virus, raise concerns about the possible consequences of coinfection with other arboviruses [9, 10]. Timely detection is essential for efficient disease control; however, in most low-income nations where the illnesses are widespread, routine screening for chikungunya is lacking. Misdiagnosis and inappropriate treatment frequently occur due to the resemblance of early symptoms to those of other febrile diseases, such as malaria, which can potentially exacerbate the severity of the condition [11, 12].

Gaining a comprehensive understanding of the frequency of chikungunya is crucial for providing accurate information for the establishment of management and treatment programmes, strategies for vaccine development, and formulation of government healthcare policies. While there have been some individual reports, the actual prevalence of the phenomenon at both national and regional levels is still uncertain [13, 14]. Therefore, it is necessary to calculate a combined prevalence estimate using meta-analysis.

Chikungunya has emerged as a notable public health issue in East Africa in recent decades [15, 16]. The disease is primarily influenced by the favorable climate and environmental circumstances in the region, which promote mosquito breeding. Several causes contribute to its high occurrence, including the process of urbanization, the effects of climate change, and insufficient methods to manage the vector [17, 18]. Urbanization results in densely populated living environments and inadequate waste disposal systems, which provide optimal circumstances for mosquito development [19]. In addition, climate change leads to variable temperatures and erratic rainfall patterns, which make it more challenging to manage mosquito populations [20].

In Kenya, the coastal districts have had repeated chikungunya epidemics, which have been made easier by the presence of many mosquitoes. The significant epidemic on Lamu Island in 2004-2005 emphasized the susceptibility of the region [21]. In a similar vein, Tanzania has had numerous instances of epidemics, when the virus has disseminated swiftly in both urban and rural regions [22]. The absence of efficient vector control programmes and the restricted public health infrastructure worsen the problem, making containment difficult [23].

Chikungunya epidemics have also occurred in Uganda, namely, in the northern regions [24]. The virus's transmission is facilitated by the movement of individuals and goods across borders, as well as the existence of refugee camps [25, 26]. These camps frequently lack sufficient sanitation and healthcare services, which makes managing outbreaks more complicated. The existence of other vector-borne illnesses, such as malaria and dengue, places additional strain on public health resources [27–29].

Despite these obstacles, there have been focused endeavors to combat chikungunya infection [30]. Various solutions, including as public awareness campaigns, improved surveillance systems, and strengthened vector control measures, have been deployed by governments and international organizations [31]. The Ministry of Health in Kenya has escalated its efforts to educate the people about preventive measures [32, 33]. In a similar vein, Tanzania has enhanced its disease surveillance systems, facilitating prompt identification and reaction to epidemics [22, 34].

Nevertheless, there are still notable deficiencies. Further extensive research is required to fully comprehend the transmission dynamics of the virus and to create efficacious vaccinations and treatments. Enhancing healthcare facilities and securing sufficient funds for vector control programmes are essential. International cooperation and assistance are crucial, as addressing chikungunya necessitates a synchronized endeavor beyond national boundaries.

An awareness and comprehension of the frequency of chikungunya in the human population will assist in the implementation of management and treatment programmes, formulation of vaccine development strategies, and formulation of healthcare policies by the government.

The national and regional prevalence of chikungunya is yet unclear [24, 33, 35], despite the existence of isolated occurrences. Therefore, a combined prevalence estimate is relevant. This systematic review and meta-analysis attempts to establish a dependable foundation of evidence regarding the geographic spread of chikungunya in the human population. This systematic review and meta-analysis aim to present accurate and fundamental data on the geographic distribution of chikungunya in East Africa. It provides the first comprehensive study of the prevalence of chikungunya in the region using a meta-analytical technique. The evidence reported here is expected to strengthen the case for doing a thorough laboratory study of febrile diseases.

## 2. Method

We performed a systematic review and meta-analysis of published studies that followed the PRISMA guidelines [36]. The focus of our study was to determine the prevalence of chikungunya in East Africa. A protocol for the study was registered in PROSPERO with ID no: CRD42024556658.

### 2.1. Search Strategy and Selection Criteria

We conducted a comprehensive search across five electronic databases, namely, Web of Science, PubMed, ScienceDirect, Scopus, and Google Scholar. Our search utilized a combination of specific keywords to address the worldwide impact of chikungunya. The search technique utilized was Chikungunya OR CHIK OR CHIKV) AND (prevalence OR seroprevalence OR burden OR epidemiology OR epidemiological OR epidemic OR endemic OR outbreak OR occurrence OR Distribution). The comprehensive search technique employed in the databases is outlined in the Supporting document (B1). The initial search was performed on February 21, 2024, and the record was subsequently updated on June 7, 2024, which marks the last search. The search was limited to publications published from 2014 onwards, with no language limitations. This study encompassed cross-sectional, prospective, and retrospective research investigating the prevalence of chikungunya in East Africa. The exclusion criteria were as follows: (1) case reports, reviews, opinions, perspectives, book chapters, and editorials; (2) nonhuman studies; (3) articles without accessible full texts; (4) studies lacking clear data on chikungunya infection; and (5) studies relying on self-reported infections rather than laboratory-confirmed diagnoses. To ensure a comprehensive search procedure, the references from the papers that were included were carefully evaluated and reviewed.

Mendeley was used to eliminate duplicate studies. Two writers (ASAM and BKE) conducted a separate evaluation of the titles and abstracts of the papers and then performed a comprehensive examination of the full text to determine which research studies were eligible. Disputes concerning inclusion were handled through deliberation and, if required, arbitration involving other writers. The study included individuals of all ages and genders who are from East Africa. The main outcome assessed was the presence of persons who were infected with chikungunya at the same time.

### 2.2. Data Extraction and Quality Assessment

Throughout the screening process, all three writers individually evaluated the complete text, abstract, and title of the chosen papers. The authors collected relevant data and arranged it in a table, resolving any inconsistencies through consensus after being evaluated by a fourth author. The screening procedure comprised three stages: title screening, abstract screening, and full-text examination. The extracted data comprised the primary author's name, year of publication, sample location, sample size, number of cases, detection technique, sample type, antibiotics employed, and study design.

In order to accurately assess the prevalence, the data with the highest resistance rate was chosen while testing various carbapenem drugs, in order to prevent any underestimation. Three authors independently employed the Joanna Briggs Institute (JBI) prevalence data appraisal guidelines (Supporting file B2) to assess the quality of the studies. The provided evaluation checklist, consisting of nine variables, sets standards for a typical research study. The results were evaluated using a coding system in which “zero (0)” indicated “NO” and “two (1)” indicated “YES.” According to the strict evaluation standards of the Joanna Briggs Institute, investigations were assigned a rating ranging from 0 to 9. In this study, studies with a score below seven (7) were considered undesirable, while those with a score of seven (7) or higher were regarded to be of good quality and included in the analysis. The quality scores for each study can be found in Supporting file B3.

### 2.3. Data Analysis

The data analysis was conducted using two software packages: Comprehensive Meta-Analysis programme and OpenMeta Analyst Version 3.1 (CEBM, 2022, Providence, RI, USA) (Biostat, Inc., 2021, Englewood, NJ, USA). The subgroup analyses considered the geographical region, research duration, and source of the samples. Given the significant differences in how samples were collected, the timeframes for collection, and the methods used for detection, the random effects model was considered the best suitable for this investigation. The meta-analysis utilized the DerSimonian and Laird method to get the combined prevalence [37]. In addition, a forest plot was generated to assess the significance, effect size, prevalence, confidence intervals (CIs), and heterogeneity among studies.

The presence of publication bias was examined using funnel plots and Egger's regression test [38]. The inconsistency index Statistics (*I*^2^) measures the level of heterogeneity at the study level. Values above 75%, 50%, and 25% indicate high, moderate, and low levels of heterogeneity, respectively [39]. The Cochrane *Q* test was used to evaluate heterogeneity, and nonsignificant heterogeneity was considered acceptable. A subgroup meta-analysis was performed to pinpoint the precise factors responsible for the variation observed in the data.

## 3. Result

### 3.1. Study Selection

**Figure 1:**
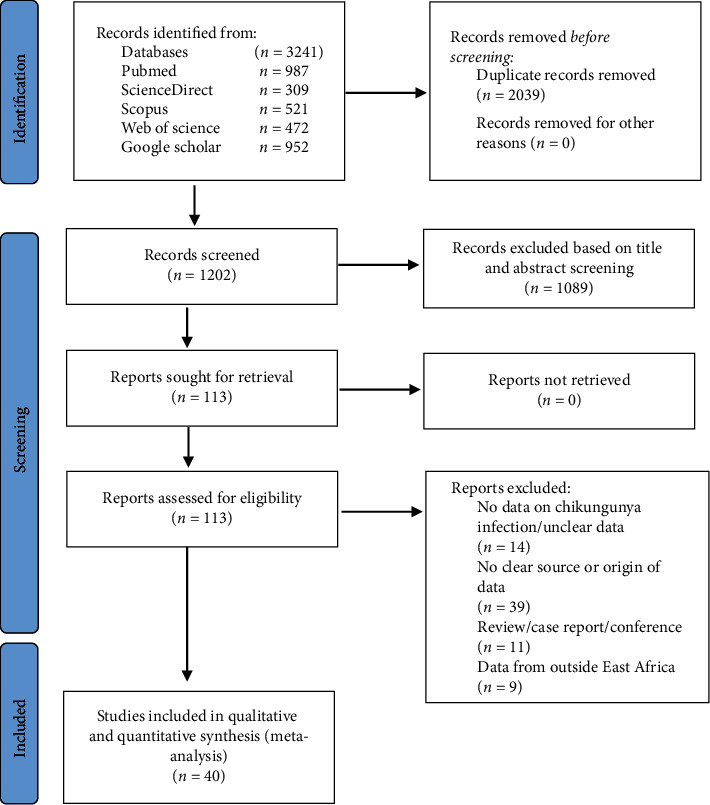
Summary of the studies selection and screening process.

We conducted a search on five electronic databases and found a total of 3214 records. Following the elimination of duplicate articles, a total of 1202 articles were left for the purpose of screening their titles and abstracts. A total of 1089 articles were excluded based on the specified exclusion criteria. Subsequently, the complete texts of the remaining articles were evaluated to determine their suitability. A further 73 papers were removed due to reasons such as duplicated data, lack of data on CHIKV infection, and ambiguous reporting of prevalence statistics. In the end, a total of 40 publications satisfied the eligibility criteria and were incorporated into the qualitative synthesis and meta-analysis.  Figure 1 provides a comprehensive overview of the research selection procedure.

### 3.2. Characteristic of the Included Studies

Research undertaken throughout East Africa between 2014 and 2024 provides valuable information about the occurrence of CHIKV. This evaluation encompassed a comprehensive analysis of 40 studies conducted across 13 nations in East Africa. Tanzania had the highest share of the studies included, with a total of 8, followed by Kenya with 6. The proportion of cross-sectional studies was the largest (*n* = 32), while the frequency of prospective research was the lowest (*n* = 1). The included studies predominantly utilized the ELISA method for detection. Especially remarkable are the studies with the largest and smallest sample sizes and the highest number of positive cases. In Kenya, Vu et al. [40] did a study with a sample size of 3835 individuals, which is the largest in terms of number. They used ELISA to discover 14 positive cases, suggesting a relatively low infection rate. In contrast, the research conducted by EMILIE (2023) in Djibouti had a small sample size of only 58 subjects. However, it revealed a surprisingly high prevalence of 57 positive cases utilizing RT-PCR. However, in Rwanda, Seruyange et al. [41] conducted a study with 874 participants and found the largest number of positive cases (551) using ELISA. This indicates a significant outbreak, as shown in Table 1. Several research studies have been undertaken in Kenya, which have provided diverse data on infection rates and detection methods. According to Waggoner et al. [32], there were no instances of positive cases detected among the 385 participants using RT-PCR. This suggests that there may have been a lesser prevalence of the condition or limits in its identification during that time. However, Inziani et al. [42] identified 36 individuals who tested positive for the virus out of a total of 656 participants using the ELISA method, indicating a significant presence of the virus. Konongoi et al. [43] documented 23 instances of the virus among 189 individuals in Kenya and Somalia by employing the ELISA technique, highlighting the virus's geographical expansion. Grossi-Soyster et al. [48] detected 141 instances of positivity out of a total of 500 participants, while Musak et al. [49] identified 92 positive cases out of 1378 individuals using ELISA/RT-PCR. These findings suggest the occurrence of substantial outbreaks, as reported in Table 1. Research conducted in Tanzania has yielded a wide range of results. In a study conducted by K. Elfving in 2016, no instances of the disease were found among the 677 participants who were tested using ELISA. This indicates that there may be variations in the frequency of outbreaks or that efficient control measures were in place during that period. Chipwaza et al. [47] detected 17 instances of positivity out of 364 individuals through the utilization of RT-PCR. Similarly, Mwanyika et al. [34] documented 509 positive cases out of 1818 participants in a cross-sectional study employing ELISA/RT-PCR, as indicated in Table 1.

Mozambique's research likewise indicates substantial prevalence rates. Muianga et al. [46] detected 46 instances of positivity out of 146 individuals by the utilization of ELISA. Similarly, Antonio et al. [51] reported 49 positive cases among 895 participants in a retrospective study employing ELISA, indicating significant levels of infection. In Sudan, Adams et al. [54] identified seven individuals who tested positive for the disease out of a total of 379 participants using the ELISA method. Similarly, Baudin et al. [55] detected 39 positive cases out of 130 participants using the RT-PCR technique, as indicated in Table 1.

### 3.3. Prevalence of Chikungunya in East Africa

**Figure 2:**
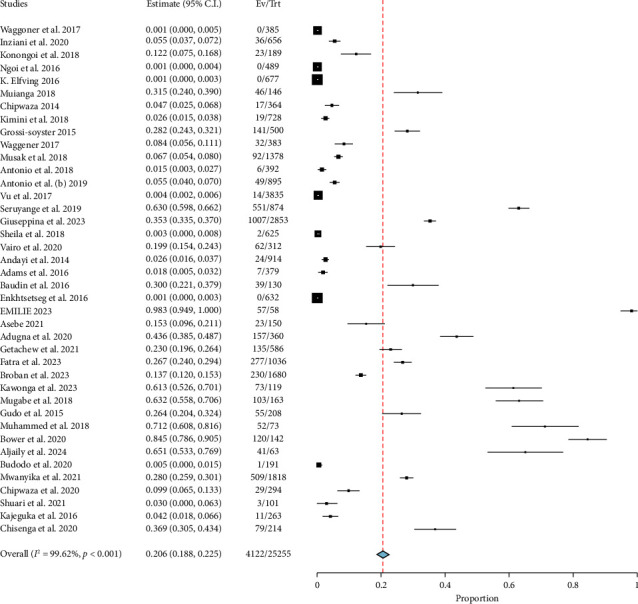
Forest plot showing pooled prevalence of chikungunya in East Africa.

**Figure 3:**
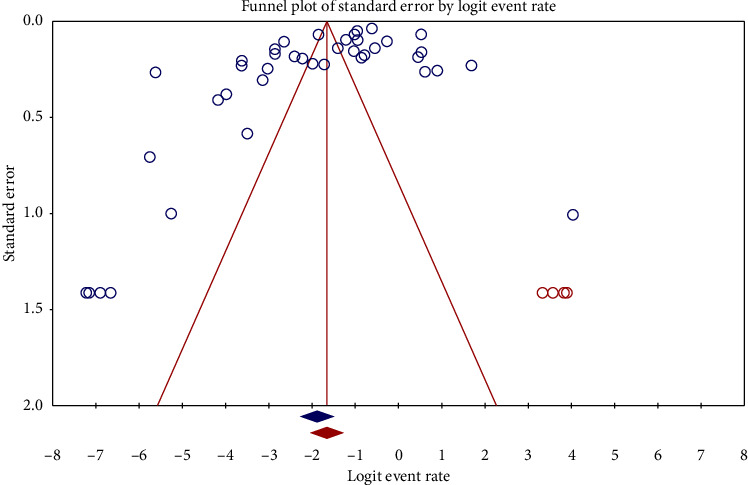
Funnel plot showing publication bias in the pooled prevalence of chikungunya in East Africa.

The prevalence of chikungunya in East Africa was found to be high, with an overall pooled estimate of 20.6% and a CI ranging from 18.8% to 22.5%. The prevalence of chikungunya in East Africa, as indicated in Figure 2, was found to be significant with a pooled prevalence rate of *p* < 0.001. In addition, there was a high amount of heterogeneity, with an *I*^2^ value of 99.62%. However, the included studies exhibited a publication bias, as indicated by the Egger's *p* (0.010) and the asymmetrical distribution of the studies in proportion to standard error, as seen in Figure 3.

### 3.4. Subgroup-Pooled Prevalence of Chikungunya in East Africa

**Figure 4:**
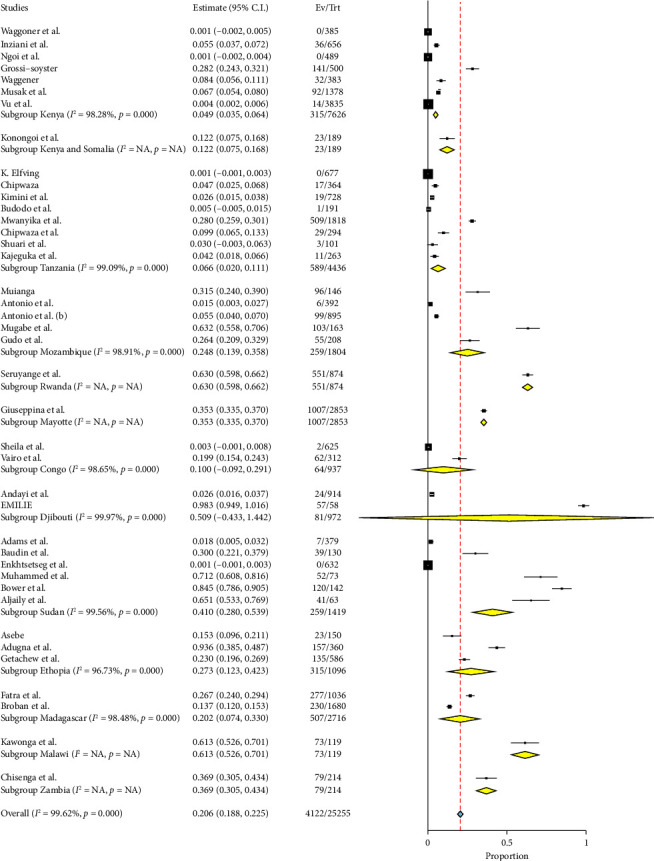
Forest plot showing the subgroup analysis of chikungunya in East Africa in relation to country.

**Figure 5:**
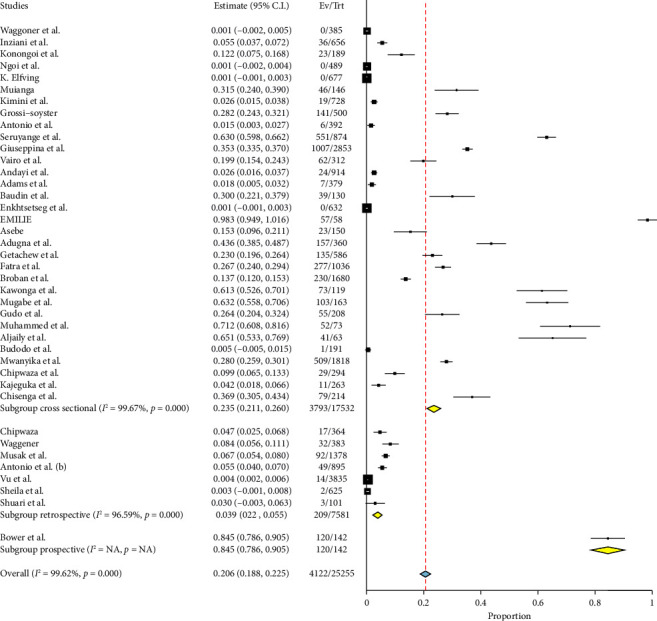
Forest plot showing the subgroup analysis of chikungunya in East Africa in relation to study designs.

**Figure 6:**
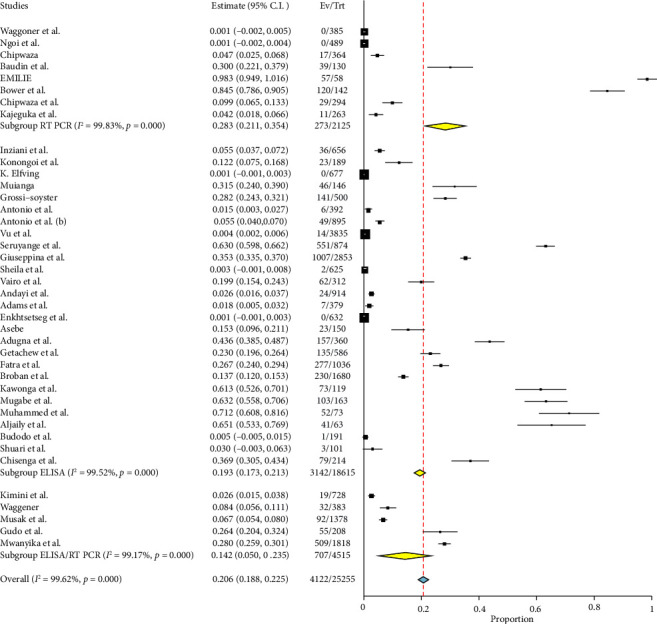
Forest plot showing the subgroup analysis of chikungunya in East Africa in relation to method of detection.

**Figure 7:**
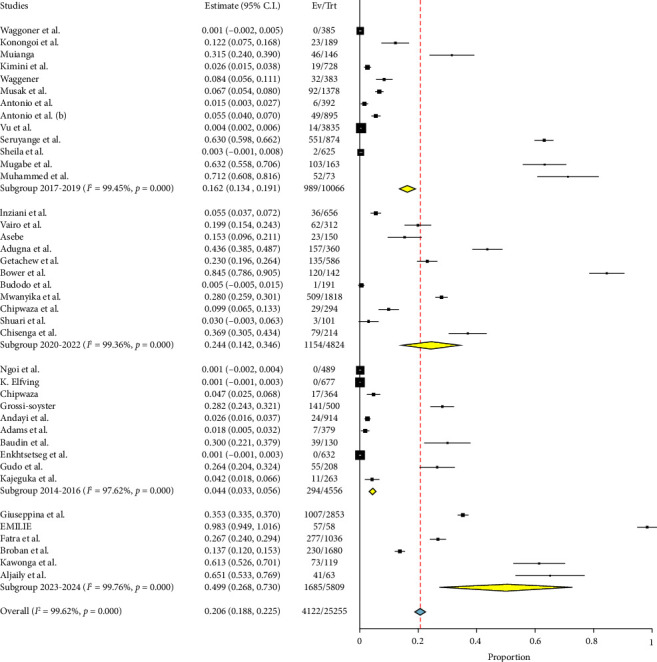
Forest plot showing the subgroup analysis of chikungunya in East Africa in relation to year of publication.

Extensive research efforts have been conducted between 2014 and 2024 to thoroughly investigate the occurrence of CHIKV in different East African countries. The rates of occurrence and measures of diversity vary greatly among various nations, research methodologies, and publication years. The specific information regarding the subgroup meta-analysis can be found in Table 2 and Figures 4, 5, 6, and 7.

#### 3.4.1. Subgroup Analysis in Relation to Country

Seven studies conducted in Kenya have reported a prevalence rate of 4.9% (95% CI: 3.5–6.4) with a high level of heterogeneity (*Q* = 348.81, *I*^2^ = 98.28%, and *p* < 0.001). This indicates a moderate prevalence of the condition with significant variation in the data, as seen in Figure 4. One study conducted in Kenya and Somalia found a higher prevalence rate of 12.2% (95% CI: 7.5–16.8), suggesting that there are variations in infection rates between different regions. Tanzania had a prevalence of 6.6% (95% CI: 2.0–11.1) based on eight research. There is also considerable heterogeneity (*Q* = 765.60, *I*^2^ = 99.09%, and *p* < 0.001), indicating significant diversity in the data (Figure 4). Mozambique has a notable prevalence rate of 24.8% (95% CI: 13.9–35.8) based on five research studies. The data also show a significant level of heterogeneity (*Q* = 365.69, *I*^2^ = 98.91%, and *p* < 0.001). A study conducted in Rwanda found a prevalence rate of 63.0% (95% CI: 59.8–66.2), indicating a notably high occurrence. Similarly, a single study conducted in Mayotte revealed a prevalence rate of 35.3% (95% CI: 33.5–37.0), indicating a significant occurrence as well (Figure 4). In Congo, two investigations were conducted, and the combined prevalence was found to be 10.0% (95% CI: 9.2–29.1), with a significant level of variation (*Q* = 74.16, *I*^2^ = 98.65%, and *p* < 0.001). Djibouti exhibits a notably high prevalence rate of 50.4% (95% CI: 43.3–144.2) based on two studies. In addition, there is a remarkably high level of heterogeneity (*Q* = 2858.02, *I*^2^ = 99.97%, and *p* < 0.001), suggesting considerable changes in the data. The prevalence of Sudan is 41.0% (95% CI: 28.0–53.9) based on six research studies. The data also indicate considerable heterogeneity (*Q* = 1128.15, *I*^2^ = 99.56%, and *p* < 0.001). In Ethiopia, three studies have reported a prevalence rate of 27.3% (95% CI: 12.3–42.3), indicating a high level of variation (*Q* = 61.22, *I*^2^ = 96.73%, and *p* < 0.001). Two investigations conducted in Madagascar have found a prevalence rate of 20.2% (95% CI: 7.4–33.0), indicating a significant level of variation (*Q* = 65.62, *I*^2^ = 98.48%, and *p* < 0.001). According to a single study, Malawi has a prevalence rate of 61.3% (95% CI: 52.6–70.1), while Zambia has a prevalence rate of 36.9% (95% CI: 30.5–43.4), as shown in Table 2.

#### 3.4.2. Subgroup Analysis in Relation to Study Designs

When analyzing several study designs, it was found that out of the 32 cross-sectional studies examined, the prevalence of the condition was 23.5% (95% CI: 21.1–26.0). These studies showed a high level of heterogeneity, with a *Q* value of 9446.18, an *I*^2^ value of 99.67%, and a *p* value of less than 0.001. Seven retrospective studies collectively indicate a reduced prevalence rate of 3.9% (95% CI: 2.2–5.5) with significant heterogeneity (*Q* = 175.71, *I*^2^ = 96.59%, and *p* < 0.001). A single study suggests that there is a remarkably high occurrence rate of 84.5% (95% CI: 78.6–90.5) (Figure 5).

#### 3.4.3. Subgroup Analysis in Relation to the Detection Method

When examining the methodologies used to identify the virus, a total of eight RT-PCR investigations found a prevalence rate of 28.3% (95% CI: 21.1–35.4). These studies also showed a significant level of heterogeneity (*Q* = 4148.87, *I*^2^ = 99.83%, and *p* < 0.001). The ELISA investigations, totaling 27, indicate a prevalence of 19.3% (95% CI: 17.3–21.3) with a significant level of heterogeneity (*Q* = 5396.62, *I*^2^ = 99.52%, and *p* < 0.001). Five studies utilizing both ELISA/RT-PCR techniques have found a prevalence rate of 14.2% (95% CI: 5.0–23.5) with significant heterogeneity (*Q* = 483.67, *I*^2^ = 99.17%, *p* < 0.001) as shown in Figure 6.

#### 3.4.4. Subgroup Analysis in Relation to Year of Publication

The publication years provide insights into the fluctuating prevalence rates over time. Research conducted between 2017 and 2019, consisting of 13 studies, indicates that the prevalence of the condition under investigation is 16.2% (with a 95% CI ranging from 13.4% to 19.1%). The data also reveal a significant level of variation among the studies (heterogeneity), with a Q value of 2180.36, an *I*^2^ value of 99.45%, and a *p* value of less than 0.001. The findings from a total of 11 studies conducted between 2020 and 2022 suggest a higher occurrence rate of 24.4% (95% CI: 14.2–34.6) with significant variability (*Q* = 1574.54, *I*^2^ = 99.36%, and *p* < 0.001). Research conducted between 2014 and 2016, consisting of 10 studies, found that the prevalence rate was 4.4% (with a 95% CI of 3.3–5.6). The data also showed a high level of heterogeneity (*Q* = 378.71, *I*^2^ = 97.62%, and *p* < 0.001). A total of six recent investigations conducted between 2023 and 2024 have revealed a remarkably high prevalence rate of 49.9% (95% CI: 26.8–73.0). The data also indicate a significant level of heterogeneity, with a Q value of 2081.92, an I^2^ value of 99.76%, and a *p* value of less than 0.001. These findings are illustrated in Figure 7.

## 4. Discussion

The prevalence of chikungunya in tropical Africa is of great importance from an epidemiological perspective [7, 69]. Despite various attempts by both governmental and nongovernmental organizations to control the vector and monitor the disease, the number of chikungunya cases in East Africa has been steadily increasing over the past 20 years [70]. This study, which involved a systematic review and meta-analysis, examines the prevalence of CHIKV across East Africa. Our study has discovered a very high occurrence of chikungunya in East Africa, with a combined prevalence rate of 20.6% (95% CI: 18.8%–22.5%). This figure highlights the significant impact on public health caused by chikungunya in the region. The statistical significance of the pooled prevalence (*p* < 0.001) suggests a strong and convincing association, which justifies the need for prompt attention and intervention. The presence of heterogeneity indicates that there are significant variances in the reported prevalence rates among various studies. These disparities can be related to changes in study design, demographic characteristics, diagnostic procedures, and temporal factors. The wide range of variations highlights the intricate nature of the epidemiology of chikungunya in East Africa. The findings of this study are in alliance with the report of others [1, 51, 71].

The results of our research align with earlier studies that have documented elevated occurrence rates of chikungunya in East African nations. The study conducted by Mboera et al. revealed that the prevalence rate of the condition in Tanzania was 22.5% [71]. Similarly, Clements et al. reported a high prevalence rate in Uganda [11]. These studies, in addition to our own findings, emphasize the widespread occurrence of chikungunya in the region and the necessity for continuous public health initiatives to reduce its effects.

The study's findings indicate a significant occurrence and diversity, which have multiple possible consequences. First and foremost, it is imperative to enhance surveillance systems to efficiently monitor and address chikungunya epidemics. Improved monitoring can aid in the prompt identification and quick intervention, therefore decreasing the overall impact of the condition. Furthermore, public health education and community participation play a vital role in increasing understanding regarding chikungunya, including its transmission and prevention strategies. This can aid in mitigating the transmission of the virus and minimizing the consequences of epidemics.

Furthermore, the variation in prevalence that has been found highlights the significance of implementing targeted public health interventions that are specifically designed to address the distinct epidemiological circumstances of each individual country. By harnessing collective data and resources, collaborative initiatives among East African countries have the potential to enhance the efficacy of control and preventative policies for CHIKV. The collaborative strategy described here is in accordance with the recommendations made by Antonio et al. [51], which emphasize the importance of regional cooperation in dealing with emerging infectious diseases. The existence of publication bias, as evidenced by the Egger's *p* value and the skewed distribution of studies in relation to the standard error, gives rise to issues regarding the dependability and accuracy of the combined prevalence estimate. Publication bias may arise when studies demonstrating significant or favorable outcomes are more prone to being published compared to those with nonsignificant or negative results [38], thus resulting in an overestimation of the actual prevalence.

The subgroup meta-analysis revealed that Tanzania and Kenya are identified as crucial regions for CHIKV research, playing a substantial role in the study pool analyzed in this review. These nations, including Djibouti, Rwanda, and Mozambique, exhibit a distinct range of CHIKV prevalence, which indicates different patterns of disease spread and public health obstacles. Mozambique has a notable prevalence, along with a high level of heterogeneity. This suggests the presence of substantial outbreak clusters or methodological inconsistencies in the studies conducted. The findings of this analysis are consistent with the report of others [46, 61]. There was variation in the prevalence of chikungunya in many East African nations, the exact cause for the variation in the sample group and the number of positive cases is not known, but these differences emphasize the diversity in the transmission patterns of CHIKV, which may be affected by local environmental and socioeconomic factors. The outcomes of this investigation are consistent with the reports of other researchers [11, 32, 33, 40].

The wide range of methodologies used in research studies that sum up this review, which mainly employed ELISA and RT-PCR for CHIKV detection, suggests different diagnostic capabilities and potentially varying levels of sensitivity and specificity among these methods, which are in alliance with the report of others [72–74]. A comparative examination of cross-sectional and retrospective studies provides a more detailed understanding of the epidemiological intricacies. The bulk of cross-sectional research studies offer a picture of prevalence at distinct moments in time, whereas the rarer retrospective and prospective studies provide limited longitudinal insights due to their lower frequency [75–80]. A standardized regional surveillance plan is required due to the different rates of occurrence and different methods used. This strategy should combine both serological and molecular diagnostic techniques to improve accuracy. Moreover, the notable outbreaks found in certain studies necessitate focused efforts on vector control, public awareness campaigns, and strengthening hospital infrastructure to reduce the transmission of CHIKV and its related morbidity. Powers emphasized the importance of implementing strong vector surveillance and control measures to effectively manage arboviral infections such as CHIKV [31].

The analysis based on study design indicates that cross-sectional studies demonstrate a very high combined prevalence, which reflects the comprehensive overview these studies offer across diverse groups. Retrospective studies indicate a reduced occurrence, possibly attributed to the inherent constraints of collecting data retrospectively, such as the influence of memory bias and insufficient documentation. The solitary prospective study suggests an unusually elevated occurrence, either due to the study's emphasis on a group at high risk or during an outbreak time. The findings of this study are concordant with the report of Mahalingaiah et al. [77].

Examining the frequency of occurrence throughout various years of publication uncovers fluctuating patterns in the epidemiology of CHIKV. Research conducted between 2014 and 2016 indicates a low occurrence; however, studies conducted between 2023 and 2024 demonstrate a notably increased occurrence. This upward trajectory may suggest an increase in the transmission of CHIKV, better identification and reporting methods, or a greater emphasis on studying regions with high frequency in recent times.

### 4.1. Strength and Limitation

A comprehensive study and meta-analysis were undertaken to ascertain the prevalence of chikungunya in East Africa. This study is the first to report the prevalence of chikungunya in East Africa. It followed the PRISMA criteria, which ensured a strong and reliable methodological framework. By doing a comprehensive search across six electronic databases and utilizing precise keywords, a detailed review of the literature was obtained, hence reducing the likelihood of overlooking significant studies. The incorporation of a wide variety of research methodologies (including cross-sectional, prospective, and retrospective studies) and the implementation of rigorous quality evaluation standards (based on the Joanna Briggs Institute recommendations) enhances the dependability of the results. The study's insights are enhanced by the utilization of two software packages for data analysis and the inclusion of numerous factors, such as geographical location, research period, and sample source, in the subgroup analyses. The study's thorough examination of 40 publications from 13 countries in East Africa, along with the creation of a forest plot to evaluate the importance, magnitude, prevalence, CIs, and variation among the research, highlights the comprehensive and rigorous nature of the investigation. Nevertheless, the study contains significant constraints. Due to a lack of sufficient data in certain countries, caution should be exercised when interpreting the results, as they may not accurately reflect the overall prevalence of chikungunya in East Africa. Furthermore, the existence of publication bias, as indicated by Egger's test and the skewed distribution of studies, raises doubts regarding the dependability and accuracy of the combined prevalence estimate. This bias may result in an overestimation of the actual prevalence. Notwithstanding these difficulties, the results of the study align with prior research, emphasizing the widespread occurrence of chikungunya in East Africa and the necessity for continuous public health initiatives to tackle this substantial health problem.

## 5. Conclusion

The status of CHIKV in East Africa is high and of epidemiological significance and stresses the need for an effective vector control and surveillance scheme in East Africa.

## Figures and Tables

**Table 1:** Characteristic of the included study in the epidemiology of chikungunya in east Africa.

Name of author	Year of publication	Country	Total sample	Positive cases	Design	Method of detection
Waggoner et al. [32]	2017	Kenya	385	0	Cross sectional	RT PCR
Inziani et al. [42]	2020	Kenya	656	36	Cross sectional	ELISA
Konongoi et al. [43]	2018	Kenya and Somalia	189	23	Cross sectional	Elisa
Ngoi et al. [44]	2016	Kenya	489	0	Cross sectional	RT PCR
Elfving et al. [45]	2016	Tanzania	677	0	Cross sectional	ELISA
Muianga et al. [46]	2018	Mozambique	146	46	Cross sectional	ELISA
Chipwaza et al. [47]	2014	Tanzania	364	17	Retrospective	RT PCR
Kimini et al. [35]	2018	Tanzania	728	19	Cross sectional	ELISA/RT PCR
Grossi-Soyster et al. [48]	2015	Kenya	500	141	Cross sectional	ELISA
Waggener et al. [32]	2017	Kenya	383	32	Retrospective	ELISA/RT PCR
Musak et al. [49]	2018	Kenya	1378	92	Retrospective	ELISA/RT PCR
Antonio et al. [50]	2018	Mozambique	392	6	Cross sectional	ELISA
Antonio et al. [51]	2019	Mozambique	895	49	Retrospective	ELISA
Vu et al. [40]	2017	Kenya	3835	14	Retrospective	ELISA
Seruyange et al. [41]	2019	Rwanda	874	551	Cross sectional	ELISA
Giuseppina et al. [52]	2023	Mayotte	2853	1007	Cross sectional	ELISA
Sheila et al. [22]	2018	Congo	625	2	Retrospective	ELISA
Vairo et al. [53]	2020	Congo	312	62	Cross sectional	ELISA
Andayi et al. [1]	2014	Djibouti	914	24	Cross sectional	ELISA
Adams et al. [54]	2016	Sudan	379	7	Cross sectional	ELISA
Baudin et al. [55]	2016	Sudan	130	39	Cross sectional	RT PCR
Enkhtsetseg et al. [56]	2016	Sudan	632	0	Cross sectional	ELISA
Emilie et al. (Emilie et al., 2023)	2023	Djibouti	58	57	Cross sectional	RT PCR
Asebe et al. [57]	2021	Ethiopia	150	23	Cross sectional	ELISA
Adugna et al. (Adugna et al., 2020)	2020	Ethiopia	360	157	Cross sectional	ELISA
Getachew et al. (Getachew et al., 2021)	2021	Ethiopia	586	135	Cross sectional	ELISA
Fatra et al. (Fatra et al., 2023)	2023	Madagascar	1036	277	Cross sectional	ELISA
Broban et al. [58]	2023	Madagascar	1680	230	Cross sectional	ELISA
Kawonga et al. [59]	2023	Malawi	119	73	Cross sectional	ELISA
Mugabe et al. [60]	2018	Mozambique	163	103	Cross sectional	ELISA
Gudo et al. [61]	2015	Mozambique	208	55	Cross sectional	ELISA/RT PCR
Muhammed et al. [62]	2018	Sudan	73	52	Cross sectional	ELISA
Bower et al. [63]	2020	Sudan	142	120	Prospective	RT PCR
Aljaily et al. [64]	2024	Sudan	63	41	Cross sectional	ELISA
Budodo et al. [65]	2020	Tanzania	191	1	Cross sectional	ELISA
Mwanyika et al. [34]	2021	Tanzania	1818	509	Cross sectional	ELISA/RT PCR
Chipwaza et al. [47]	2020	Tanzania	294	29	Cross sectional	RT PCR
Shuari et al. [66]	2021	Tanzania	101	3	Retrospective	ELISA
Kajeguka et al. [67]	2016	Tanzania	263	11	Cross sectional	RT PCR
Chisenga et al. [68]	2020	Zambia	214	79	Cross sectional	ELISA

**Table 2:** Subgroup meta-analysis of chikungunya in East Africa in relation to the country of origin, study designs, method of detection, and year of publication.

Parameter	Number of studies	Prevalence (%)	Confidence interval	*Q*	*I* ^2^ (%)	Heterogeneity	
						DF	*p*
Country							
Kenya	7	4.9	3.5–6.4	348.81	98.28	6	< 0.001
Kenya and Somalia	1	12.2	7.5–16.8	—	—	—	—
Tanzania	8	6.6	2.0–11.1	765.60	99.09	7	< 0.001
Mozambique	5	24.8	13.9–35.8	365.69	98.91	4	< 0.001
Rwanda	1	63.0	59.8–66.2	—	—	—	—
Mayotte	1	35.3	33.5–37.0	—	—	—	—
Congo	2	10.0	9.2–29.1	74.16	98.65	1	< 0.001
Djibouti	2	50.4	43.3–144.2	2858.02	99.97	1	< 0.001
Sudan	6	41.0	28.0–53.9	1128.15	99.56	5	< 0.001
Ethiopia	3	27.3	12.3–42.3	61.22	96.73	2	< 0.001
Madagascar	2	20.2	7.4–33.0	65.62	98.48	1	< 0.001
Malawi	1	61.3	52.6–70.1	—	—	—	—
Zambia	1	36.9	30.5–43.4	—	—	—	—
Study designs							
Cross sectional	32	23.5	21.1–26.0	9446.18	99.67	31	< 0.001
Retrospective	7	3.9	2.2–5.5	175.71	96.59	6	< 0.001
Prospective	1	84.5	78.6–90.5	—	—	—	—
Method of detection							
RT-PCR	8	28.3	21.1–35.4	4148.87	99.83	7	< 0.001
ELISA	27	19.3	17.3–21.3	5396.62	99.52	26	< 0.001
ELISA/RT PCR	5	14.2	5.0–23.5	483.67	99.17	4	< 0.001
Year of publication							
2017–2019	13	16.2	13.4–19.1	2180.36	99.45	12	< 0.001
2020–2022	11	24.4	14.2–34.6	1574.54	99.36	10	< 0.001
2014–2016	10	4.4	3.3–5.6	378.71	97.62	9	< 0.001
2023-2024	6	49.9	26.8–73.0	2081.92	99.76	5	< 0.001

## Data Availability

The dataset supporting the conclusions of this manuscript is available from the corresponding author upon reasonable request.

## Supporting Information

Supporting Information B1: Search strategy of the study.

Supporting Information B2: JBI checklist for prevalence studies.

Supporting Information B3: Quality assessment of the included studies.
