# Supplementary material for: Current Epidemiological Status of Chikungunya Virus Infection in East Africa: A Systematic Review and Meta-Analysis
Source: J Trop Med. 2024 Oct 25;2024:7357911. doi: 10.1155/2024/7357911 (PMC11530290; doi:10.1155/2024/7357911)
Supplement: Supporting Information — B1: Search strategy of the study. [file 7357911.f1.pdf]

PUBMED

Search: **((((((((((((((((((((Chikunugunya OR chikunguya))) AND (Kenya)) OR (Somalia)) OR (Rwanda)) OR (Burundi)) OR (Ethiopia)) OR (seychelles)) OR (Djibouti)) OR (madagascar)) OR (Malawi)) OR (Mauritius)) OR (Eritrea)) OR (Zambia)) OR (Reunion)) OR (Uganda)) OR (Mozambique)) OR (Sudan)) OR (Zimbabwe)) OR (Tanzania)) OR (Comoros)) OR (Mayotte)**
("chikunguya"[All Fields] AND ("kenya"[MeSH Terms] OR "kenya"[All Fields] OR "kenya s"[All Fields])) OR ("somalia"[MeSH Terms] OR "somalia"[All Fields] OR "somalia s"[All Fields]) OR ("rwanda"[MeSH Terms] OR "rwanda"[All Fields] OR "rwanda s"[All Fields]) OR ("burundi"[MeSH Terms] OR "burundi"[All Fields]) OR ("ethiopia"[MeSH Terms] OR "ethiopia"[All Fields] OR "ethiopia s"[All Fields]) OR ("seychelles"[MeSH Terms] OR "seychelles"[All Fields]) OR ("djibouti"[MeSH Terms] OR "djibouti"[All Fields]) OR ("madagascar"[MeSH Terms] OR "madagascar"[All Fields] OR "madagascar s"[All Fields]) OR ("malawi"[MeSH Terms] OR "malawi"[All Fields] OR "malawi s"[All Fields]) OR ("mauritius"[MeSH Terms] OR "mauritius"[All Fields]) OR ("eritrea"[MeSH Terms] OR "eritrea"[All Fields]) OR ("zambia"[MeSH Terms] OR "zambia"[All Fields] OR "zambia s"[All Fields]) OR ("reunion"[MeSH Terms] OR "reunion"[All Fields] OR "reunions"[All Fields]) OR ("uganda"[MeSH Terms] OR "uganda"[All Fields] OR "uganda s"[All Fields]) OR ("mozambique"[MeSH Terms] OR "mozambique"[All Fields] OR "mozambique s"[All Fields]) OR ("sudan"[MeSH Terms] OR "sudan"[All Fields] OR "sudans"[All Fields] OR "sudan s"[All Fields]) OR ("zimbabwe"[MeSH Terms] OR "zimbabwe"[All Fields] OR "zimbabwe s"[All Fields]) OR ("tanzania"[MeSH Terms] OR "tanzania"[All Fields] OR "tanzania s"[All Fields]) OR ("comoros"[MeSH Terms] OR "comoros"[All Fields] OR "comoro"[All Fields]) OR ("comoros"[MeSH Terms] OR "comoros"[All Fields] OR "mayotte"[All Fields])

Translations

Kenya: "kenya"[MeSH Terms] OR "kenya"[All Fields] OR "kenya's"[All Fields]
Somalia: "somalia"[MeSH Terms] OR "somalia"[All Fields] OR "somalia's"[All Fields]
Rwanda: "rwanda"[MeSH Terms] OR "rwanda"[All Fields] OR "rwanda's"[All Fields]
Burundi: "burundi"[MeSH Terms] OR "burundi"[All Fields] OR "burundi's"[All Fields]
Ethiopia: "ethiopia"[MeSH Terms] OR "ethiopia"[All Fields] OR "ethiopia's"[All Fields]
seychelles: "seychelles"[MeSH Terms] OR "seychelles"[All Fields]
Djibouti: "djibouti"[MeSH Terms] OR "djibouti"[All Fields]
madagascar: "madagascar"[MeSH Terms] OR "madagascar"[All Fields] OR "madagascar's"[All Fields]
Malawi: "malawi"[MeSH Terms] OR "malawi"[All Fields] OR "malawi's"[All Fields]
Mauritius: "mauritius"[MeSH Terms] OR "mauritius"[All Fields]
Eritrea: "eritrea"[MeSH Terms] OR "eritrea"[All Fields] OR "eritrea's"[All Fields]
Zambia: "zambia"[MeSH Terms] OR "zambia"[All Fields] OR "zambia's"[All Fields]

Reunion: "reunion"[MeSH Terms] OR "reunion"[All Fields] OR "reunions"[All Fields]
Uganda: "uganda"[MeSH Terms] OR "uganda"[All Fields] OR "uganda's"[All Fields]
Mozambique: "mozambique"[MeSH Terms] OR "mozambique"[All Fields] OR "mozambique's"[All Fields]
Sudan: "sudan"[MeSH Terms] OR "sudan"[All Fields] OR "sudans"[All Fields] OR "sudan's"[All Fields]
Zimbabwe: "zimbabwe"[MeSH Terms] OR "zimbabwe"[All Fields] OR "zimbabwe's"[All Fields]
Tanzania: "tanzania"[MeSH Terms] OR "tanzania"[All Fields] OR "tanzania's"[All Fields]
Comoros: "comoros"[MeSH Terms] OR "comoros"[All Fields] OR "comoro"[All Fields]
Mayotte: "comoros"[MeSH Terms] OR "comoros"[All Fields] OR "mayotte"[All Fields]

Scopus
(chikungunya) AND (virus) AND (prevalence)

Google Scholar
allintitle: Chikungunya prevalence OR OR OR seroprevalence OR OR OR distribution OR OR OR occurence "chikungunya virus"

Web of Science
(chikungunya) AND (virus) AND (prevalence)

Science Direct
chikungunya virus infection prevalence
