# Supplementary material for: Current Epidemiological Status of Chikungunya Virus Infection in East Africa: A Systematic Review and Meta-Analysis
Source: J Trop Med. 2024 Oct 25;2024:7357911. doi: 10.1155/2024/7357911 (PMC11530290; doi:10.1155/2024/7357911)
Supplement: Supporting Information — B3: Quality assessment of the included studies. [file 7357911.f3.pdf]

QUALITY OF INCLUDED STUDIES BY JBI CRITICAL APPRAISAL CHECKLIST FOR STUDIES REPORTING PREVALENCE DATA

S/N	Name of authors [reference] and year of publication		JBI checklist*									Total
1	Waggoner et al. (2017) [32]	2017	1	2	3	4	5	6	7	8	9	
2	Inziani et al. (2020) [41]	2020	Yes	No	Yes	Yes	Yes	Yes	Yes	Yes	Yes	8
3	Konongoi et al. (2018) [42]	2018	Yes	No	Yes	Yes	Yes	Yes	Yes	Yes	Yes	8
4	Ngoi et al. (2016) [43]	2016	Yes	No	Yes	Yes	Yes	Yes	Yes	Yes	Yes	8
5	Elfving et al [44]	2016	Yes	No	Yes	Yes	Yes	Yes	Yes	Yes	Yes	8
6	Muianga et al. [45]	2018	Yes	No	Yes	Yes	Yes	Yes	Yes	Yes	Yes	8
7	Chipwaza et al.[46]	2014	Yes	No	Yes	Yes	Yes	Yes	Yes	Yes	Yes	8
8	Kimini et al [47]	2018	Yes	No	Yes	Yes	Yes	Yes	Yes	Yes	Yes	8
9	Grossi-soyster et al[48]	2015	Yes	No	Yes	Yes	Yes	Yes	Yes	Yes	Yes	8
10	Waggener et al[32]	2017	Yes	No	Yes	Yes	Yes	Yes	Yes	Yes	Yes	8
11	Musak et al [49]	2018	Yes	No	Yes	Yes	Yes	Yes	Yes	Yes	Yes	8
12	Antonio et al [50]	2018	Yes	No	Yes	Yes	Yes	Yes	Yes	Yes	Yes	8
13	Antonio et al. [51]	2019	Yes	No	Yes	Yes	Yes	Yes	Yes	Yes	Yes	8
14	Vu et al [52]	2017	Yes	No	Yes	Yes	Yes	Yes	Yes	Yes	Yes	8
15	Seruyange et al [53]	2019	Yes	No	Yes	Yes	Yes	Yes	Yes	Yes	Yes	8
16	Giuseppina et al[54]	2023	Yes	No	Yes	Yes	Yes	Yes	Yes	Yes	Yes	8
17	Sheila et al (Sheila et al., 2018)	2018	Yes	No	Yes	Yes	Yes	Yes	Yes	Yes	Yes	8
18	Vairo et al [56]	2020	Yes	No	Yes	Yes	Yes	Yes	Yes	Yes	Yes	8
19	Andayi et al [1]	2014	Yes	No	Yes	Yes	Yes	Yes	Yes	Yes	Yes	8
20	Adams et al [57]	2016	Yes	No	Yes	Yes	Yes	Yes	Yes	Yes	Yes	8
21	Baudin et al [58]	2016	Yes	No	Yes	Yes	Yes	Yes	Yes	Yes	Yes	8
22	Enkhtsetseg et al [59]	2016	Yes	No	Yes	Yes	Yes	Yes	Yes	Yes	Yes	8
23	Emilie et al (Emilie et al., 2023)	2023	Yes	No	Yes	Yes	Yes	Yes	Yes	Yes	Yes	8
24	Asebe et al [61]	2021	Yes	No	Yes	Yes	Yes	Yes	Yes	Yes	Yes	8
25	Adugna et al (Adugna et al., 2020)	2020	Yes	No	Yes	Yes	Yes	Yes	Yes	Yes	Yes	8
26	Getachew et al (Getachew et al., 2021)	2021	Yes	No	Yes	Yes	Yes	Yes	Yes	Yes	Yes	8
27	Fatra et al (Fatra et al., 2023)	2023	Yes	No	Yes	Yes	Yes	Yes	Yes	Yes	Yes	8
28	Broban et al [65]	2023	Yes	No	Yes	Yes	Yes	Yes	Yes	Yes	Yes	8
29	Kawonga et al [66]	2023	Yes	No	Yes	Yes	Yes	Yes	Yes	Yes	Yes	8
30	Mugabe et al [67]	2018	Yes	No	Yes	Yes	Yes	Yes	Yes	Yes	Yes	8
31	Gudo et al [68]	2015	Yes	No	Yes	Yes	Yes	Yes	Yes	Yes	Yes	8
32	Muhammed et al [69]	2018	Yes	No	Yes	Yes	Yes	Yes	Yes	Yes	Yes	8
33	Bower et al [70]	2020	Yes	No	Yes	Yes	Yes	Yes	Yes	Yes	Yes	8
34	Aljaily et al [71]	2024	Yes	No	Yes	Yes	Yes	Yes	Yes	Yes	Yes	8
35	Budodo et al [72]	2020	Yes	No	Yes	Yes	Yes	Yes	Yes	Yes	Yes	8
36	Mwanyika et al [34]	2021	Yes	No	Yes	Yes	Yes	Yes	Yes	Yes	Yes	8
37	Chipwaza et al [46]	2020	Yes	No	Yes	Yes	Yes	Yes	Yes	Yes	Yes	8
38	Shuari et al [73]	2021	Yes	No	Yes	Yes	Yes	Yes	Yes	Yes	Yes	8
39	Kajeguka et al [74]	2016	Yes	No	Yes	Yes	Yes	Yes	Yes	Yes	Yes	8
40	Chisenga et al [75]	2020	Yes	No	Yes	Yes	Yes	Yes	Yes	Yes	Yes	8

JBI CHECKLIST* 1. Appropriate sampling frame to address target population, **2.** Appropriate sampling way of study participants, **3.** Adequate sample size, **4.** Detail description of study participants and settings, **5.** Data analysis with sufficient coverage of identified sample, **6.** Use of valid methods to identify the condition, **7.** Standard, reliable way of measurement of condition for all participants, **8.** Availability of appropriate statistical analysis, **9.** Adequate response rate and management of low response rate.

Scores are coded as Yes=1 and No=0.
